# Supplementary material for: Humans store about 1.5 megabytes of information during language acquisition
Source: R Soc Open Sci. 2019 Mar 27;6(3):181393. doi: 10.1098/rsos.181393 (PMC6458406; doi:10.1098/rsos.181393)
Supplement: Table of assumptions used in our estimates [file rsos181393supp1.pdf]

Table S1. The assumptions we make in our estimates.

Section	Domain	Assumptions
(a)	Phonemes	1. The language system must contain information about acoustic cues to phoneme identity.<br>2. The maximum entropy over the frequency dimension can be represented as a uniform distribution over audible frequency ranges.<br>3. The maximum entropy over the VOT dimension can be represented as a uniform distribution ranging from -200 to 200 ms.<br>4. The variance in language users' representations of acoustic cues for phonemes can be well approximated by normal distributions following [12].
(b)	Phonemic Wordforms	1. The language system favors compression of statistical co-occurrences.<br>3. The cost of specifying a language model over phonemes is negligible.<br>4. Adult language users have a lexicon of $40,000$ lexical entries.<br>5. The sample of words we used to induce our estimate is an adequate approximation to the adult lexicon.
(c)	Lexical Semantics	1. Semantic space can be represented as a multivariate normal distribution with independent dimensions.<br>2. The maximum entropy over the space can be approximated by a normal distribution whose standard deviation is the maximum distance between words.<br>3. What learners come to know about the semantics of words narrows the distribution over semantic space based on distance to the nearest semantic neighbor.<br>4. Adult language users have a lexicon of $40,000$ lexical entries.<br>5. Our sample of words is a decent approximation to the distances of the average word.
(d)	Word Frequency	1. Errors in word frequency discrimination are a result of insufficient representational resolution.<br>2. Subjective frequency rankings are well approximated by objective frequency rankings (via corpus statistics).<br>3. Adult language users have a lexicon of $40,000$ lexical entries.<br>4. The sample of words we used in our experiment are representative of the words in the adult lexicon.
(e)	Syntax	1. The language system must contain information to uniquely identify one binary parse tree from all possible binary parse trees.<br>2. The maximum entropy over syntactic parses is given by the number of binary parse trees.<br>3. Sentences from [39] are a good approximation/coverage of the essentially independent syntactic components of English grammar.

Dear Editors,

Thank you for great news! We were real pleased with the reviewers' comments and are happy to submit the manuscript in the requested format.

Thank you for considering Royal Society Open Science as an outlet for your work. Two reviewers and I have read your manuscript, and we are all of the opinion that it provides an interesting argument and a provocative platform for more debate. I am happy to recommend that the manuscript be accepted as it is. If you would like to make the adjustment that Reviewer 1 highlights, you are welcome to, but I leave that decision up to you.

We have slightly changed the abstract and last paragraph to clarify/simplify our position. With regard to Review 1, we did not change the manuscript but respond below.

Thank you.

Best,
Frank Mollica & Steve Piantadosi

The ms is exceptionally well written. As it happens, I have only one issue. It is the estimate of word frequency at the bottom of p. 7. Using a factor of two to get the upper limit would give a limit of 4 bits rather than 3, no? Wouldn't change much in terms of conclusions, but in my understanding would be more correct.

We introduce the factor of error on the estimate of the mental representations of the bins not the bit estimates themselves. So our best guess 4 bins = log2(4) = 2 bits per word. Lower bounded as 2 bins = log2(2) = 1 bit per word. Upper bounded as 8 bins = log2(8) = 3 bits per word.
